# Use of quantum hyperlight technology in photobiomodulation on stem cells: an experimental in vitro study

**DOI:** 10.1007/s10103-025-04358-2

**Published:** 2025-02-15

**Authors:** Gülsemin Çiçek, Fatma Öz Bağcı, Tahsin Murad Aktan, Selçuk Duman

**Affiliations:** Department of Histology and Embryology, Faculty of Medicine, Necmettin Erbakan University, Konya, Turkey

**Keywords:** Mesenchymal stem cell, Hyperglycemia, Quantum hyperlight, Fullerene, Cellular therapy

## Abstract

Human umbilical cord matrix Wharton’s jelly mesenchymal stem cells (WJ-MSCs) are commonly utilized in regenerative medicine due to their therapeutic benefits. However, the microenvironmental stress present in patients with hyperglycemia can significantly reduce mesenchymal stem cell (MSC) viability under high-glucose conditions in the body, ultimately reducing their therapeutic effectiveness. Enhancing the survival rate of MSCs following cell transplantation remains a crucial challenge. This study investigates whether Quantum Hyperlight (QHL) can counteract the detrimental effects of high glucose (HG), thereby improving MSC survival, proliferation, and mitochondrial function. We aimed to evaluate the effect of QHL on cellular viability, proliferation, and mitochondrial activity in WJ-MSCs exposed to HG. MSCs were cultured in a medium containing normal glucose (NG) (1 g/L) and HG (4.5 g/L). MSCs in the HG medium were exposed to QHL for 90 s or 180 s with an energy density of 2.4 Joules/cm^2^/minute and an average power density of 40 mW/cm^2^. Then, proliferating cell nuclear antigen (PCNA), MTT assays, and Mitotracker Green staining were performed to evaluate cell viability and proliferation. The viability of MSCs was significantly increased in the QHL-treated groups (84% in QHL-90 s and 86% in QHL-180 s) compared to the untreated HG group (65%, *p* < 0.001). PCNA expression in QHL-90 s and QHL-180 s groups showed significant increases (*p* < 0.001) compared to the untreated HG group. MitoTracker staining intensity was significantly higher in the QHL-treated groups compared to the untreated HG group (*p* < 0.001). The HG environment reduced viability, proliferation, and mitochondrial staining. In the context of the NG environment, MSCs exhibited notable differences. However, the viability, proliferation, and mitochondrial staining rates of MSCs were significantly higher in the HG conditions when treated with QHL compared to the group that did not receive QHL. This study introduces QHL as a novel approach to enhance the therapeutic potential of WJ-MSCs under HG conditions, demonstrating its ability to improve cellular viability, proliferation, and mitochondrial activity. This study highlights its potential as a pretreatment to improve clinical outcomes in regenerative medicine.

## Introduction

Mesenchymal stem cells (MSCs) are capable of proliferation, self-renewal, and differentiation into various cell types under laboratory conditions. Stem cells are used in many clinical areas such as bone marrow transplants for the treatment of leukemia, skin transplants for burn patients, corneal transplants to restore vision, and regenerative therapies for cartilage repair. They can be isolated from various sources, including bone marrow, adipose tissue, peripheral blood, Wharton's jelly of the umbilical cord, the synovium, and other tissues. MSCs have the ability to differentiate into several cell types, such as osteoblasts, chondrocytes, adipocytes, hepatocytes, neurons, and glial lineages [1, 2]. The Mesenchymal and Tissue Stem Cell Committee of the International Society for Cellular Therapy proposes minimal criteria to define human MSC. First, MSC must be plastic-adherent when maintained in standard culture conditions. Second, MSC must express CD105, CD73 and CD90, and lack expression of CD45, CD34, CD14 or CD11b, CD79α or CD19 and HLA-DR surface molecules [3, 4]. Third, MSC must differentiate to osteoblasts, adipocytes and chondroblasts in vitro [2].

Glucose plays a critical role in regulating the fate and behavior of stem cells by influencing their proliferation, self-renewal capacity, and susceptibility to senescence. While some research indicates that high glucose (HG) concentrations can impair cellular functions and trigger apoptosis in stem cells, other studies suggest that HG may promote proliferation and enhance osteogenic differentiation potential in MSCs [5–7]. Some studies found that preconditioning MSCs with glucose increased their antidiabetic potential [8, 9]. In patients with diabetes, circulating levels of MSCs are lower, leading to inadequate movement of MSCs to the site of injury. This deficiency hampers the regeneration and repair of the affected tissue. For instance, the diabetic microenvironment diminishes the effectiveness of MSC infusion in treating osteoporosis [10, 11]. To address this, the present study models a diabetic microenvironment by exposing MSCs to HG concentrations, investigating whether Quantum Hyperlight Technology (QHL) can mitigate the detrimental effects of hyperglycemia on MSC viability, proliferation, and mitochondrial function.

**Fig. 1:**
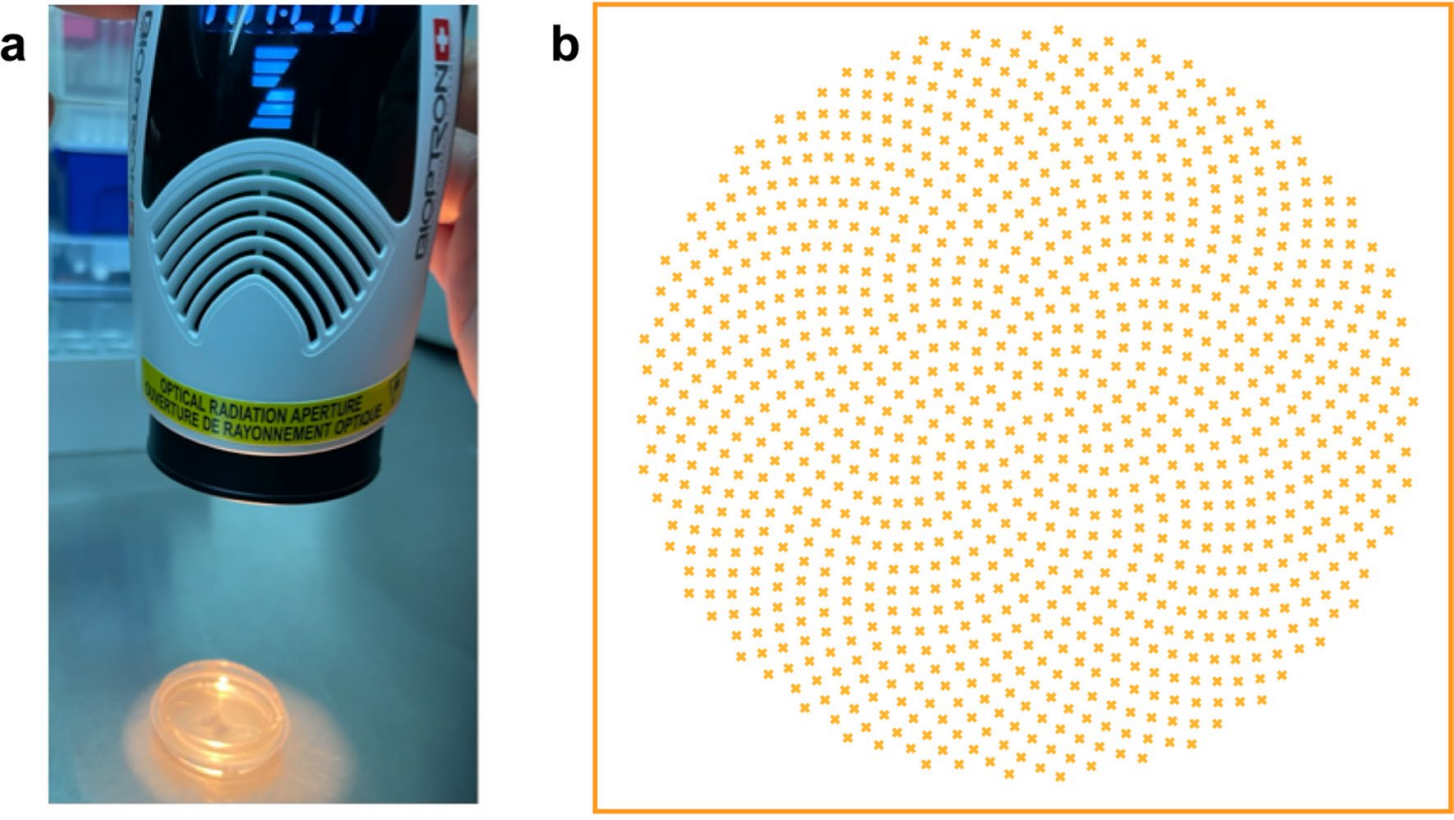
Application of polarized polychromatic incoherent light and visualization of the quantum sunflower photon pattern. **A** The experimental setup demonstrates the application of polarized polychromatic incoherent light to Wharton’s Jelly Mesenchymal Stem Cells (WJ-MSCs). **B** Visualization of the unique "sunflower photon pattern" produced by Quantum Hyperlight (QHL). This pattern reflects the distinct quantum-structured photon arrangement achieved through fullerene-based filtering, enhancing light-matter interaction at the cellular level. The pattern represents a critical feature of QHL’s mechanism of action, which may contribute to its observed therapeutic effects on MSCs

Fullerenes, stable carbon forms like C60, are recognized for their structural integrity and antioxidant properties, making them valuable in biomedical fields. C60-filtered QHL interacts with photons to produce a unique quantum "sunflower photon pattern," governed by Fibonacci's Law [12, 13] (Fig. 1b). This pattern, distinct from linearly polarized light, aligns with QHL principles to achieve biocompatibility. QHL device (Bioptron AG, Wollerau, Switzerland) device emits low-energy, polychromatic, and polarized light (480–3400 nm) with a specific energy density of 40 mW. Photons penetrate tissues up to 2.5 cm, initiating chain reactions in photosensitive biomolecules, stimulating secondary cellular responses beyond the treated area [14–16]. Food and Drug Administration (FDA) approved for pain management, QHL exhibits anti-inflammatory and immunomodulatory effects, reducing pro-inflammatory cytokines, enhancing fibroblast proliferation, and regulating lymphocyte activity [17]. Studies highlight QHL's quantum-driven therapeutic benefits in musculoskeletal pain, dermatological disorders, and systemic inflammation, reinforcing its potential as a quantum-based light therapy [18].

Photobiomodulation (PBM) is a non-invasive therapy that employs low-level light, usually in the red or near-infrared range, to influence cellular functions, enhance tissue repair, minimize inflammation, and reduce oxidative stress by activating mitochondrial pathways and boosting cellular bioenergetics. It has been reported that PBM has biostimulatory effects on the proliferation rates of keratinocytes and fibroblasts and increases collagen biosynthesis [19]. In a complete medium containing 17 mMol/L D-glucose, studies reported reduced cell migration, increased apoptosis, DNA damage, and decreased expression of interleukin-6 (IL-6) and basic fibroblast growth factor. Conversely, exposure to PBM stimulated cell migration, enhanced IL-6 expression, increased cell proliferation, and improved cell viability [20, 21]. In a study applying PBM to MSCs, it was shown to have the potential to promote MSC proliferation, differentiation, and growth factor secretion [22]. Unlike traditional PBM techniques, QHL introduces a quantum-structured photon arrangement that aligns with biological systems, potentially offering superior therapeutic benefits. However, whether MSCs preconditioned with a high-glucose medium would show these effects with PBM has not been investigated. Although different PBM methods have been used before as pretreatment for MSC culture; there are no studies using the QHL source [23, 24].

This study introduces QHL as a novel PBM approach for enhancing MSC function under hyperglycemic stress. The novelty of this study lies in its focus on how QHL influences mitochondrial function, viability, and proliferation in MSCs cultured in a diabetic-like high-glucose (HG) environment. We hypothesize that QHL exposure will counteract hyperglycemia-induced mitochondrial dysfunction, enhance MSC survival, and promote cellular proliferation, making it a promising preconditioning strategy for stem cell-based therapies in diabetic and metabolic disorders.

## Materials and methods

### Mesenchymal stem cell culture and experimental design

Human umbilical cord matrix Wharton’s Jelly Mesenchymal Stem Cells (WJ-MSCs) were commercially obtained from ATCC (PCS-500–010, Manassas, USA) and cultured in 25 cm^2^ dishes with Dulbecco’s Modified Eagle Medium (DMEM, Gibco, USA) supplemented with 10% fetal bovine serum (FBS) and 100 U/mL of penicillin/streptomycin. The NG condition contained 5.5 mM glucose*,* while the HG condition contained 25 mM glucose at 37 °C in a 5% CO2 environment. Once the cells reached 80% confluence, they were passaged. Passages 3 to 5 (P3–P5) of MSCs were used in all experiments to ensure consistency in cell behavior. After detachment from the culture dish, cells were washed and resuspended at a concentration of 1 × 10⁶ cells/mL. Flow cytometry analysis was performed following established protocols for MSC characterization. Briefly, MSCs were incubated for 30 min with CD146, CD90, CD105, and CD45 monoclonal antibodies (R&D Systems, USA) at 4 °C in the dark. After incubation, an anti-mouse IgG phycoerythrin conjugate was added for 20 min, followed by washing with phosphate-buffered saline (PBS). A total of 10,000 gated events were recorded per sample using CytoFLEX Flow Cytometry (Beckman Coulter, USA) and analyzed with CytExpert software.

In our study, the cells were categorized into four experimental groups.:

- Group 1: Group 1: Cells were cultured in DMEM supplemented with 10% FBS and 100 U/mL of penicillin/streptomycin, containing 1 g/L (5.5 mM) glucose, which represents normal physiological glucose levels;
- Group 2: Cells passaged with culture medium containing HG (4.5 g/L; 25 mM).
- Group 3: Cells passaged with culture medium containing HG (4.5 g/L; 25 mM) and exposed to QHL for ninety seconds every day.
- Group 4: Cells passaged with culture medium containing HG (4.5 g/L; 25 mM) and exposed to QHL for one hundred eighty seconds every day.

Group 1 (NG) served as the negative control, representing MSCs cultured under physiological glucose conditions without high-glucose-induced stress or QHL treatment. Group 2 (HG, untreated) functioned as the experimental control, allowing assessment of the detrimental effects of HG on MSCs in the absence of QHL treatment. Group 1 provided our control group. As the HG dose, we used 4.5 g/L, which is referenced in the literature [25, 26]. The HG group was specifically chosen to mimic conditions found in diabetic patients, where hyperglycemia significantly impacts MSC functionality. The study's objective was to evaluate the potential of QHL in reversing these negative effects, which is why no additional NG group exposed to light was included.

All experiments were conducted at least three repetitions (n ≥ 3) for each experimental condition to ensure reliability and reproducibility of the results.

### Treatment of the MSCs cultures with QHL

A Bioptron® MedAll (Bioptron AG, Wollerau, Switzerland) equipped with a nanophotonic fullerene filter was used to irradiate the cells (Table 1). The QHL device was selected for its ability to emit a broad spectrum of low-energy light, specifically targeting cellular chromophores while avoiding ultraviolet radiation. Cell culture samples were exposed to QHL at a 90-degree right angle from an 80 mm distance for 90 or 180 s (Fig. 1a). The depth of the nutrient solution exposed to light was 5 mm in all experiments. The light was applied at the same time every day for 4 days. MTT (3-(4,5-dimethylthiazol-2-yl)−2,5-diphenyltetrazolium bromide) assay, proliferating cell nuclear antigen (PCNA) expression, and mitochondrial staining were conducted at the end of the 4-day QHL treatment period to evaluate the cumulative effects of repeated exposure rather than an immediate response to a single session.

**Table 1:** Hyperpolarized polychromatic incoherent light parameters

Parameter	Value
Wavelength	350–3400 nm
Degree of polarization	> 95% (590–1550 nm)
Specific power density	av. 40 mW/cm^2^
Light energy per minute	av. 2.4 J/cm^2^
Light Intensity	min. 10.000 lx
Power Supply	100–240 V ~ 50/60 Hz
Filter / Glass diameter	ap. 5 cm

### Proliferating cell nuclear antigen assay

PCNA is in the nucleus and serves as an indicator of proliferation potential during the G1 and S phases of the cell cycle. PCNA is a nuclear protein primarily involved in DNA replication and repair. However, under conditions of cellular stress, apoptosis, or necrosis, nuclear proteins, including PCNA, can be released into the extracellular space. Previous studies have reported the detection of nuclear proteins in culture supernatants because of cell turnover or apoptosis. In this study, PCNA levels were measured in the culture medium to assess potential cell damage or release under different experimental conditions. PCNA levels in the culture media were analyzed using a sandwich enzyme-linked immunosorbent (ELISA) kit (Nepenthe, Kocaeli, Türkiye) based on assay ELISA technology. The kit involved pre-coating 96-well plates with anti-PCNA antibodies. Standards, test samples, and biotin-conjugated detection antibodies were added to the wells and washed with a wash buffer. HRP-Streptavidin was introduced, and unbound conjugates were removed through additional washes. The HRP enzymatic reaction produced a blue-colored product that transitioned to yellow. The optical density absorbance was measured at 450 nm, allowing for the calculation of PCNA concentration using a microplate reader (MultiSkan™, Thermo Scientific) USA).

### MTT assay

The viability of MSCs in vitro was assessed using the MTT assay (CyQUANT™ MTT Cell Viability Assay Kit, Invitrogen, Thermo Fisher Scientific Inc., Oregon, USA). Passage-adapted cells were plated in 96-well culture plates at a density of 10,000 cells per well. MTT reagent (10 µL of the 12-mM) was added to each well at a volume of 10 µL per 100 µL of culture medium, followed by a 4-h incubation at 37 °C in a dark environment. After incubation, the formazan crystals were dissolved in 50 µL of dimethyl sulfoxide (DMSO). The optical density was measured at 570 nm using a microplate reader (MultiSkan™, Thermo Fisher Scientific, USA).

### Mito tracker staining

The cells were placed on coverslips for observation. Mitochondrial staining was performed using MitoTracker Green FM (Invitrogen, USA), a fluorescent dye that selectively accumulates in active mitochondria. Cells were incubated with 20 nM MitoTracker Green FM for 30 min at 37 °C. After incubation, cells were fixed with 4% paraformaldehyde and washed with PBS before imaging with an Olympus BX63 confocal microscope (Japan). Fluorescence intensity was quantified using ImageJ software, on mitochondrial assessment in stem cells*.* All four groups of MSCs were incubated with DMEM containing 20 nM of MitoTracker Green for a duration of 30 min.

Photographs were captured using a confocal microscope (Olympus BX63, Japan). The images were analyzed with ImageJ (https://imagej.nih.gov/ij/) to convert them into numerical data reflecting mitochondrial staining intensity. All samples were assessed using a consistent threshold, and the percentages of the staining area were used for the measurements.

### Statistical analysis

Statistical analyses were conducted using IBM SPSS Statistics version 29.0.0.0. All experiments were performed with at least three repetitions (n ≥ 3) for each experimental condition to ensure the reliability of the results. Data are presented as mean ± standard deviation (SD). Descriptive data are presented as absolute numbers (n) and percentages (%). One-way ANOVA was employed to identify significant differences between groups, followed by the Bonferroni multiple comparisons test. A p-value of less than 0.05 was considered statistically significant.

## Results

### Characterization of stem cells

**Fig. 2:**
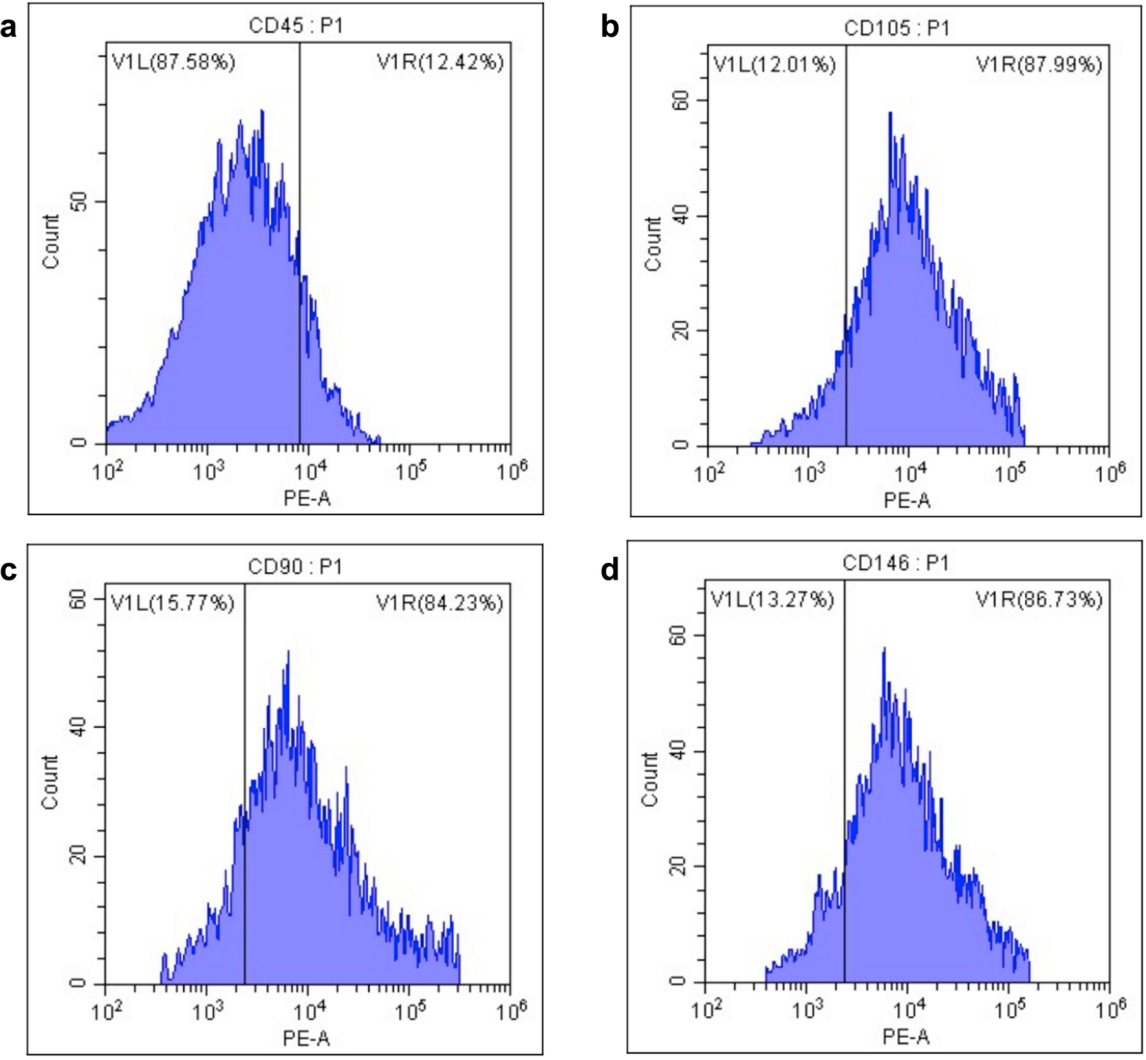
Characterization of Wharton’s Jelly Mesenchymal Stem Cells (WJ-MSCs) was performed using flow cytometry to assess the expression of specific cell surface markers. The results confirm the adherence of WJ-MSCs to the minimal criteria for mesenchymal stem cells as defined by the International Society for Cellular Therapy (ISCT). Positivity rates for each marker are as follows: **A** CD45**:** 12.42%, **B** CD105: 87.99%, **C** CD90: 84.23%, **D** CD146: 86.73%, a marker related to perivascular origin and multipotency. These results confirm the identity of WJ-MSCs as a highly pure mesenchymal cell population, exhibiting high positivity for MSC markers (CD105, CD90, and CD146) and low expression of the hematopoietic marker CD45

MSCs exhibited a characteristic fusiform and spindle-shaped phenotype within 24 h of seeding, indicating their typical morphology and adherence properties. This observation is consistent with the well-established morphology of MSCs during in vitro culture. Surface marker analysis performed via flow cytometry confirmed the identity of MSCs by demonstrating high positivity for canonical MSC markers: CD90 (84.23%), CD146 (86.73%), and CD105 (87.99%), while showing negligible expression of the hematopoietic marker CD45 (12.42%). These results align with the minimal criteria set forth by the International Society for Cellular Therapy (ISCT) for defining MSCs. These findings establish the multipotent nature of the MSCs used in this study and support their potential for applications in regenerative medicine (Fig. 2).

### PCNA assay results

**Fig. 3:**
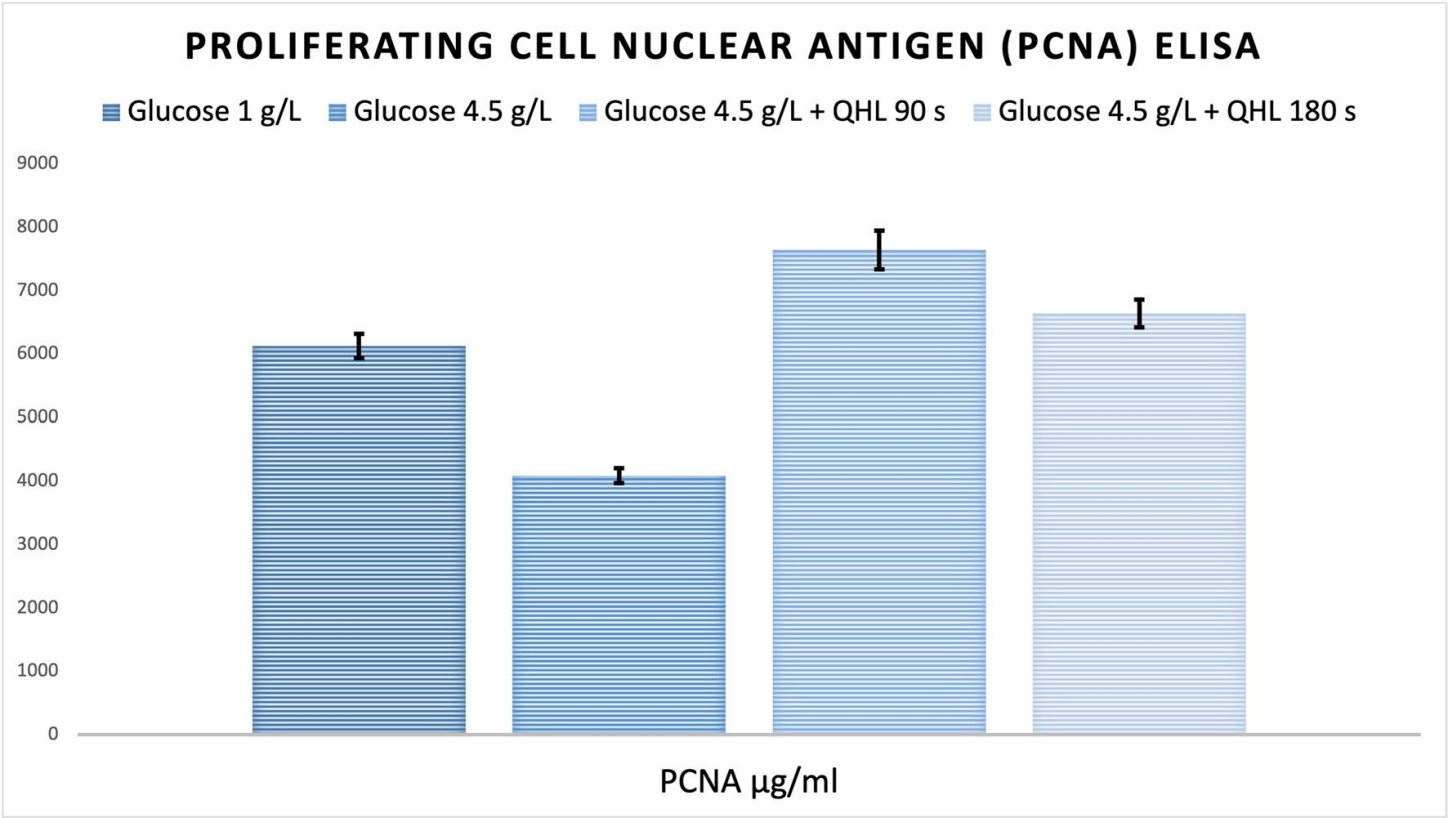
PCNA expression in Wharton’s Jelly Mesenchymal Stem Cells (WJ-MSCs) cultured under different glucose conditions and treated with Quantum Hyperlight (QHL). Group 1 (NG) represents normal glucose (1 g/L); Group 2 (HG) represents high glucose (4.5 g/L); Groups 3 and 4 (HG + QHL-90 s, HG + QHL-180 s) indicate high-glucose MSC cultures exposed to QHL for 90 or 180 s per day, respectively. PCNA levels were significantly lower in Group 2 compared to Group 1 (*p* < 0.001), whereas QHL treatment significantly restored PCNA expression in Groups 3 and 4 compared to Group 2 (*p* < 0.001). Data are presented as mean ± standard deviation (SD) from three independent experiments (*n* = 3), analyzed using one-way ANOVA with Bonferroni post hoc test

PCNA results, which are shown in Fig. 3, provide important insights into the proliferation dynamics of MSCs under varying experimental conditions. PCNA, a marker associated with cell proliferation and DNA replication, was evaluated in the culture supernatants of MSCs. The statistical analysis revealed significant differences across the experimental groups when analyzed using a Bonferroni post hoc test. Specifically, Group 2 (HG, untreated) exhibited significantly lower PCNA levels compared to Group 1 (NG) (*p* < 0.001). This reduction in PCNA expression is consistent with the well-documented negative impact of hyperglycemia on MSC proliferation, as high-glucose environments are known to induce cellular stress, impair metabolic activity, and reduce mitotic capacity. In contrast, Groups 3 and 4, which were exposed to QHL for 90 s and 180 s, respectively, demonstrated a significant increase in PCNA levels compared to Group 2 (*p* < 0.001 for both). The increase in PCNA expression observed in QHL-treated groups suggests that QHL has a mitigating effect on the inhibitory influence of hyperglycemia. Specifically, the enhancement of PCNA levels indicates improved MSC proliferation and a restoration of cell cycle activity in the presence of HG.

### MTT assay results

**Fig. 4:**
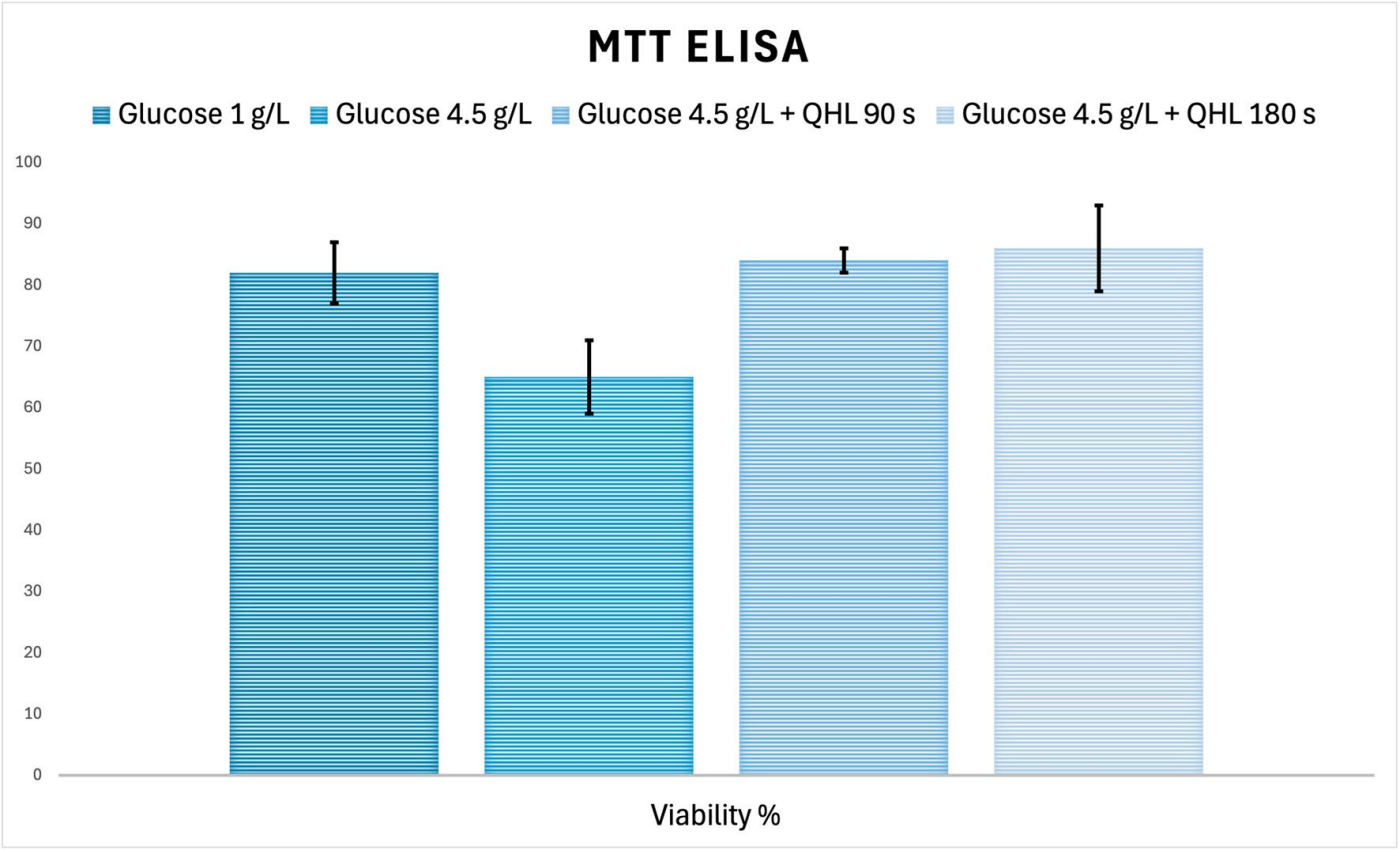
MTT Assay Results for Wharton’s Jelly Mesenchymal Stem Cells (WJ-MSCs) Groups. MTT absorbance results showing the viability of WJ-MSC under different glucose and treatment conditions. Experimental groups include: Group 1 (Normal glucose(NG): 1 g/L), Group 2 ( High glucose (HG): 4.5 g/L), Group 3 (HG + Quantum hyperlight (QHL) 90 s), and Group 4 (HG + QHL 180 s). The results indicate a significant reduction in cell viability in the Group 2 compared to the normal-glucose group (Group 1, *p* = 0.001). Treatment with QHL significantly restored cell viability in Groups 3 and 4 compared to Group 2 (*p* < 0.001 for both comparisons). Notably, no significant difference in viability was observed between Groups 3 and 4 (*p* = 1.00), suggesting that both exposure durations (90 s and 180 s) were equally effective. Statistical analysis was performed using one-way ANOVA followed by Bonferroni post hoc testing

The cellular viability results, depicted in Fig. 4, illustrate the impact of different experimental conditions on the metabolic activity and survival of MSCs. Cell viability was assessed using the MTT assay, which evaluates mitochondrial metabolic activity as an indicator of viable cells. Statistical analyses were performed using a Bonferroni post hoc test to identify significant differences between groups. In Group 2 (HG, untreated), the mean cellular viability was measured at 65%, which was significantly lower compared to Group 1 (NG), where the viability reached 82% (*p* = 0.001). In contrast, Groups 3 and 4, which were exposed to QHL for 90 s and 180 s daily, respectively, demonstrated a significant improvement in cellular viability compared to Group 2 (*p* < 0.001 for both comparisons). The mean viability values were 84% in Group 3 and 86% in Group 4, indicating that QHL effectively mitigates the adverse effects of high-glucose conditions. There was no significant difference in viability between Groups 3 and 4 (*p* = 1.00), suggesting that extending QHL exposure from 90 to 180 s did not yield additional benefits in this specific context.

### Mito tracker staining results

**Fig. 5:**
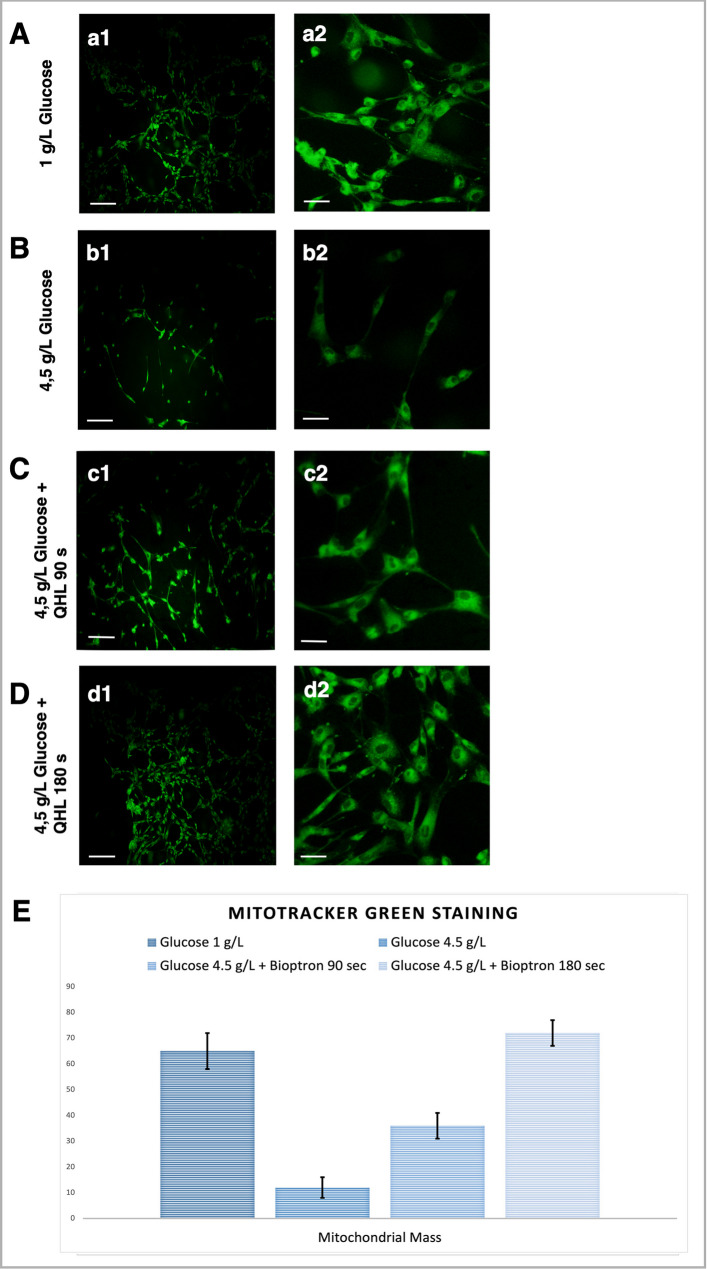
Mitochondrial mass in Wharton’s Jelly Mesenchymal Stem Cells (WJ-MSCs) was evaluated using MitoTracker Green FM staining and visualized with confocal microscopy. Mitochondria appear as green fluorescence in all images, indicating active mitochondrial function. Images a1-d1 represent lower magnification (scale bar: 100 μm), while images a2-d2 show higher magnification (scale bar: 25 μm) for detailed visualization of mitochondrial structures in the experimental groups: Group 1 (**a1**, **a2**): Normal glucose(NG) (1 g/L). Group 2 (**b1**, **b2**): High glucose(HG) (4.5 g/L). Group 3 (**c1**, **c2**): HG + Qunatum Hyperlight (QHL) 90 s). Group 4 (**d1**, **d2**): HG + QHL 180 s). Panel (**E**) presents a quantitative analysis of mitochondrial mass across experimental groups. Fluorescence intensity was measured using ImageJ software, and the results were expressed as mean ± standard deviation (SD) from three independent experiments. Statistical analysis revealed a significant reduction in mitochondrial mass in Group 2 compared to Group 1 (*p* < 0.001). Mitochondrial mass significantly increased in Groups 3 and 4 compared to Group 2 (*p* < 0.001 for both comparisons), with Group 4 showing a higher mitochondrial mass than Group 3 (*p* < 0.001). These results suggest that QHL effectively restores mitochondrial function and mass in MSCs exposed to hyperglycemic conditions

Mitochondrial activity was assessed using MitoTracker Green FM, a fluorescent dye that selectively stains active mitochondria by accumulating in response to their membrane potential. Fluorescent images were captured at 10X and 40X magnifications using a fluorescence microscope, as shown in Fig. 5. These images provided qualitative and quantitative insights into the mitochondrial staining density across the experimental groups. Statistical analyses were conducted using a Bonferroni post hoc test to compare mitochondrial activity between groups. In Group 2 (HG, untreated), mitochondrial staining density was significantly reduced compared to Group 1 (NG) (*p* < 0.001). In contrast, Groups 3 and 4, which were treated with QHL for 90 s and 180 s daily, respectively, exhibited a significant improvement in mitochondrial staining density compared to Group 2 (*p* < 0.001 for both comparisons).

Specifically: Group 3 (HG + QHL-90 s): Mitochondrial staining density increased by approximately 25% compared to Group 2. Group 4 (HG + QHL-180 s): Mitochondrial staining density increased by approximately 35% compared to Group 2, indicating a dose-dependent effect of QHL on mitochondrial activity. Furthermore, mitochondrial staining in Group 4 was significantly higher than in Group 3 (*p* < 0.001), suggesting that prolonged QHL exposure enhances mitochondrial function to a greater extent.

## Discussion

To increase the therapeutic efficacy of MSCs, approaches are needed to improve their survival, proliferation, and differentiation in implanted tissues. In previous studies, MSCs exposed to laser-emitting diodes (LED) showed improved viability, proliferation, differentiation, cell metabolism, and secretion of angiogenic factors compared with non-irradiated MSCs [27, 28]. QHL spans a broad spectrum (480–3400 nm) and differs from traditional light sources due to its incoherent, out-of-phase waves and "quantum-tuned" sunflower photon pattern governed by Fibonacci principles [29]. Unlike laser or LED-based systems, QHL excludes ultraviolet radiation, ensuring safer application without risks of DNA damage, while its unique design enhances biocompatibility and biological interactions [30, 31]. QHL is utilized in clinical applications, including the management of musculoskeletal disorders, enhancement of wound healing, and modulation of inflammatory responses [32, 33].

The viability of MSCs was assessed using the MTT assay, following standard protocols as described in recent studies evaluating MSC survival under different culture conditions [34–36]. Green PBM has been demonstrated to enhance cell survival, mitigate apoptosis, alleviate oxidative stress, and suppress inflammation [37]. A study investigating the effects of low-level laser therapy (LLLT) on the viability of human dermal fibroblasts cultured in a high-glucose (HG) medium found that LLLT significantly improved cellular viability [38]. Similarly, in our study, HG conditions adversely affected MSC viability, highlighting the detrimental impact of hyperglycemia on cellular functions. However, QHL effectively counteracted these effects, significantly improving both MSC viability and proliferation rates.

Mitochondria, as central regulators of metabolism and energy production, play a pivotal role in cellular health. PBM has been shown to activate chromophores located in the mitochondrial membrane through diverse signaling pathways, thereby directly influencing mitochondrial function [39, 40]. Previous research demonstrated that high-glucose exposure modulates genes involved in insulin secretion and diabetes prevention in MSCs derived from the decidua basalis of the human placenta [9]. Consistent with these findings, our study revealed a marked reduction in mitochondrial activity, as evidenced by decreased mitochondrial staining, under HG conditions. Notably, the application of QHL for 180 s restored mitochondrial staining levels to those observed in the control group cultured under NG conditions. NG condition was set at 1 g/L (5.5 mM), which is within the physiological range for human plasma glucose levels, rather than using DMEM Low Glucose or DMEM HG formulations. Furthermore, a moderate increase in mitochondrial staining was observed with 90 s of QHL exposure, suggesting a dose-dependent effect.

QHL exposure appears to enhance mitochondrial function by promoting mitochondrial biogenesis and improving metabolic efficiency. Previous studies suggest that PBM can activate key intracellular pathways related to mitochondrial dynamics and energy production, particularly by influencing the cytochrome c oxidase (COX) enzyme in the electron transport chain. COX activation leads to increased ATP production, a reduction in oxidative stress, and enhanced mitochondrial membrane potential [41]. Additionally, PBM has been shown to modulate reactive oxygen species (ROS) signaling, which can activate the PI3K/Akt pathway, promoting cell survival, proliferation, and mitochondrial homeostasis. Furthermore, QHL's potential effects on Nrf2-mediated antioxidant responses could mitigate oxidative stress in MSCs exposed to HG. This mechanism is particularly relevant in a hyperglycemic microenvironment, where mitochondrial dysfunction is commonly associated with increased production and impaired cellular metabolism. The observed increase in MitoTracker staining in QHL-treated groups suggests that QHL may enhance mitochondrial biogenesis via the PGC-1α pathway, a key regulator of mitochondrial dynamics and energy metabolism [40, 42]. While these mechanisms align with previous studies on PBM, further research using gene expression analysis and functional assays is needed to confirm the specific molecular pathways activated by QHL in MSCs. QHL appears to enhance metabolic activity by increasing mitochondrial functionality in MSCs. We propose that this method could significantly improve the clinical therapeutic potential of polarized polychromatic noncoherent light, particularly in in vivo applications such as wound healing and angiogenesis. By accelerating regeneration and recovery processes on a mitochondrial basis, QHL represents a promising tool for advancing regenerative medicine.

Although the present study demonstrates the beneficial effects of QHL on MSC viability and mitochondrial function in vitro, in vivo conditions are inherently more complex. MSC behavior in vivo is influenced by factors such as inflammatory cytokines, extracellular matrix interactions, and paracrine signaling, which are not fully replicated in a controlled in vitro system [43]. Additionally, in a physiological environment, the availability of oxygen, glucose fluctuations, and interactions with immune cells could modify the extent and duration of QHL’s effects.Moreover, the bioavailability and penetration depth of QHL in tissues remain important factors in determining its therapeutic efficacy. While our study used direct exposure to QHL in a monolayer culture, in vivo applications would require an evaluation of tissue penetration depth, optimal exposure duration, and the effect on different cell populations within a tissue microenvironment [44].Future in vivo studies should explore whether QHL can improve MSC engraftment and functional integration into host tissues, particularly in diabetic or ischemic models where mitochondrial dysfunction is a key challenge. Investigating QHL’s impact on angiogenesis, tissue repair, and immunomodulation in animal models will be essential to validate its translational potential for regenerative medicine [45].

Our findings align with previous studies demonstrating the beneficial effects of PBM on MSCs. Prior research has shown that LLLT and other PBM modalities can enhance MSC proliferation, viability, and mitochondrial activity under various stress conditions [22, 28]. A study demonstrated that LLLT enhances fibroblast viability and reduces apoptosis in high-glucose environments, which parallels our findings that QHL treatment counteracts hyperglycemia-induced MSC dysfunction. However, unlike LLLT, QHL operates across a broader spectrum (480–3400 nm) and is filtered through fullerene nanomaterials, which may provide additional biological effects [38]. Furthermore, while existing studies have demonstrated the general benefits of PBM on cell survival, our study specifically investigates the application of QHL in a diabetic-like hyperglycemic environment, which has not been previously reported. By showing that QHL treatment improves MSC viability and proliferation under high-glucose stress, this study suggests that QHL may be a promising preconditioning strategy for MSC-based therapies in diabetic wound healing, tissue regeneration, and other hyperglycemia-related disorders. Future investigations should compare QHL with conventional LLLT or LED-based PBM to determine its relative efficacy and underlying molecular mechanisms.

This study primarily focuses on the effects of a high-glucose environment on MSCs, as hyperglycemia is a key factor in diabetic pathophysiology. A low-glucose condition was not included in the experimental design, as our aim was to assess whether QHL could counteract the adverse effects of HG rather than examine the effects of varying glucose concentrations. Future studies incorporating a broader range of glucose conditions will be necessary to provide further insights into the metabolic regulation of MSCs.

In summary, the high-glucose diabetic microenvironment reduces the proliferation, migration, and functional capacity of MSCs. However, MSCs in HG conditions can retain these properties and provide additional clinical advantages when PBM is applied. In this study, we investigated whether QHL, a novel light source not previously tested on WJ-MSCs, is effective and whether the duration of light exposure affects MSCs. QHL effectively enhances cellular metabolism, proliferation, and mitochondrial function.

The primary limitation of this study is its in vitro design, which may not fully replicate the complexity of in vivo microenvironments. Additionally, the lack of detailed optimization of light parameters limits the generalizability of the findings. Future research is needed to explore the detailed biological mechanisms and broad therapeutic potential of QHL. These findings suggest that pretreatment methods may enhance the survival and secretory effects of MSCs in clinical applications, enabling cell culture products to provide safe and effective therapies.

## Data Availability

No datasets were generated or analysed during the current study.
